# The Role of Systemic Inflammation in Age-Related Macular Degeneration Subtypes: Exploring Novel Biomarkers

**DOI:** 10.3390/diagnostics16081144

**Published:** 2026-04-11

**Authors:** Barbaros Hayrettin Unlu, Ceren Durmaz Engin, A. Taylan Ozturk

**Affiliations:** 1 Department of Ophthalmology, Menemen State Hospital, Izmir 35660, Turkey; 2 Department of Ophthalmology, Izmir Democracy University, Buca Seyfi Demirsoy Training and Research Hospital, Izmir 35390, Turkey; cerendurmaz@gmail.com; 3 Department of Ophthalmology, Izmir Tınaztepe University, Izmir 35400, Turkey; ataylan6@yahoo.com

**Keywords:** age-related macular degeneration, HALP index, pan-immune–inflammation value (PIV), systemic immune–inflammation index (SII), systemic inflammation response index (SIRI), systemic inflammation

## Abstract

**Background/Objectives**: This study aimed to compare hematological and inflammatory markers among patients with dry and wet age-related macular degeneration (AMD) and healthy controls, and to evaluate the influence of geographic atrophy (GA) in dry AMD and treatment response (TR) in wet AMD on these markers. **Methods**: The study included patients with dry AMD (*n* = 54), wet AMD (*n* = 53), and age- and sex-matched controls (*n* = 55). Hematological parameters, serum albumin, and systemic inflammatory indices, including neutrophil-to-lymphocyte ratio (NLR), platelet-to-lymphocyte ratio (PLR), systemic immune–inflammation index (SII), systemic inflammation response index (SIRI), pan-immune–inflammation value (PIV), and hemoglobin, albumin, lymphocyte, and platelet index (HALP), were compared among the groups. **Results**: Age and sex distributions did not differ significantly between groups. Compared to controls, the wet AMD group had significantly higher neutrophil counts (*p* = 0.013), red cell distribution width (RDW) (*p* = 0.033), and inflammatory indices, including NLR, PLR, SII, SIRI, and PIV (all *p* < 0.01). HALP levels were significantly lower in wet AMD (*p* < 0.001). Dry AMD patients also had higher PLR (*p* = 0.045) and RDW (*p* = 0.005) than controls. When comparing wet and dry AMD groups directly, SIRI (*p* = 0.041) and PIV (*p* = 0.029) were significantly elevated in wet AMD, indicating stronger systemic inflammatory burden. In the dry AMD subgroup, patients with GA had significantly lower hemoglobin (*p* = 0.002) and erythrocyte counts (*p* = 0.039) than those without GA. No significant differences were observed between TR-positive and TR-negative wet AMD patients. **Conclusions**: Patients with wet AMD exhibit a more pronounced systemic inflammatory profile than both dry AMD patients and healthy controls. These findings support the hypothesis that systemic inflammation may contribute to AMD pathogenesis. Geographic atrophy in dry AMD may also be associated with additional hematologic alterations, whereas treatment response in wet AMD is not reflected in systemic markers.

## 1. Introduction

Age-related macular degeneration (AMD) is a leading cause of progressive vision loss in older adults and is classified as either dry (non-exudative) or wet (exudative) [1]. Dry AMD begins with drusen accumulation beneath the retina and may progress to geographic atrophy (GA), involving loss of the photoreceptors, outer retina, and choriocapillaris. Wet AMD, in contrast, is characterized by choroidal neovascularization, leading to retinal fluid accumulation, hemorrhage, and fibrosis. The pathogenesis of AMD remains incompletely understood; however, genetic predisposition, oxidative stress, chronic inflammation, and abnormalities in the extracellular matrix have been implicated [2]. Inflammatory mechanisms are particularly thought to play a key role in disease progression. Drusen, a hallmark of early AMD, contains components such as immunoglobulins, complement proteins, and acute-phase reactants. Moreover, immune cells, including multinucleated giant cells and leukocytes, along with their cytokines, have been associated with retinal and choroidal degeneration in advanced AMD [3].

Peripheral blood-based inflammatory markers have gained increasing attention due to their non-invasive nature, reproducibility, and accessibility through routine laboratory testing. Inflammatory indices derived from complete blood count (CBC) parameters, such as the neutrophil-to-lymphocyte ratio (NLR), platelet-to-lymphocyte ratio (PLR), systemic immune–inflammation index (SII), and red cell distribution width (RDW), have been investigated as potential biomarkers in various ocular diseases. For instance, Ozgonul et al. [4] reported elevated NLR and PLR levels in patients with primary open-angle glaucoma. Similarly, increased SII and RDW levels have been observed in individuals with keratoconus, dry eye disease, and retinal vein occlusion [5,6,7]. More recently, systemic inflammatory markers have been explored in the context of AMD. While Pinna et al. [8] found no significant association between NLR, PLR, or systemic inflammation response index (SIRI) and late-stage AMD, other studies have reported elevated NLR levels in patients with the neovascular (wet) form of the disease [9,10]. These findings collectively support further investigation into the role of systemic inflammation in AMD pathophysiology.

In light of these findings, the present study aimed to evaluate a panel of systemic inflammatory biomarkers, including NLR, PLR, SIRI, SII, pan-immune–inflammation value (PIV), and hemoglobin, albumin, lymphocyte, and platelet (HALP) index, in patients with both dry and wet forms of AMD. Additionally, within the dry AMD group, patients with GA were compared to those without to investigate potential hematologic differences associated with disease progression. In the wet AMD group, inflammatory markers were also analyzed in relation to treatment response (TR).

## 2. Materials and Methods

This study was conducted according to the principles outlined in the Declaration of Helsinki and received approval from the institutional Ethics Committee. Informed consent was waived due to the retrospective nature of this study. We retrospectively reviewed the medical records of patients diagnosed with AMD at our tertiary referral center between January 2022 and January 2025. Patients were eligible for inclusion if they had CBC and serum albumin measurements obtained within one month before or after the date of ophthalmic examination. For patients with neovascular AMD, only hematological measurements obtained at the time of diagnosis and prior to the initiation of anti-VEGF therapy were included in the analysis. Individuals with coexisting ocular pathologies, inflammatory eye diseases, malignancies, hematological disorders, autoimmune conditions, a history of corticosteroid or other treatments that could potentially alter hematologic parameters or abnormal leukocyte and platelet counts, defined as leukocytosis (>10 × 10^3^ cells/mm^3^), leukopenia (<4 × 10^3^ cells/mm^3^), thrombocytosis (>450 × 10^3^ cells/mm^3^), and thrombocytopenia (<150 × 10^3^ cells/mm^3^), were excluded, in accordance with established reference ranges in the literature [11].

A total of 162 participants were included in the study. The cohort was divided into three groups: patients with dry-type AMD (*n* = 54), wet-type AMD (*n* = 53), and age- and sex-matched healthy controls (*n* = 55). Patient classification was performed at the patient level. To ensure homogeneous study groups, only individuals with bilateral involvement of the same phenotype were included. Patients with asymmetric findings between eyes (e.g., dry AMD in one eye and neovascular AMD or a normal fellow eye) were excluded from the study.

Retinal imaging data, including optical coherence tomography (DRI OCT Triton; Topcon Inc., Tokyo, Japan) and fundus fluorescein angiography, were retrospectively reviewed. The dry-type AMD group, characterized by the presence of drusen or GA in at least one eye, was further stratified into two subgroups: those with GA (*n* = 22) and those without (*n* = 32). GA was defined as complete retinal pigment epithelium and outer retinal atrophy (cRORA). This subtype is characterized on OCT by ≥250 µm of increased choroidal transmission with corresponding RPE loss and overlying photoreceptor degeneration, in the absence of an RPE tear [12]. Patients in the wet-type AMD group exhibited macular neovascularization and received anti-vascular endothelial growth factor (anti-VEGF) therapy administered monthly for three consecutive months. Based on treatment response, these patients were further categorized as responders (*n* = 29) or non-responders (*n* = 24). A reduction in central retinal thickness of less than 25% from baseline following anti-VEGF therapy was defined as non-response [13]. The control group comprised individuals scheduled for cataract surgery who underwent preoperative CBC and serum albumin testing and had no clinical signs of AMD.

Hematological parameters, serum albumin levels, and systemic inflammatory indices, including NLR, PLR, SII, SIRI, PIV, and HALP, were analyzed. The formulas for these indices were as follows: NLR = neutrophils/lymphocytes; PLR = platelets/lymphocytes; SII = (neutrophils × platelets)/lymphocytes; SIRI = (neutrophils × monocytes)/lymphocytes; PIV = (neutrophils × platelets × monocytes)/lymphocytes; and HALP = (hemoglobin × albumin × lymphocytes)/platelets.

Statistical analyses were performed using SPSS Statistics version 28 (IBM Corp., Armonk, NY, USA). The normality of data distribution was assessed using the Kolmogorov–Smirnov test. For normally distributed variables, results were expressed as mean ± standard deviation, whereas non-normally distributed variables were presented as median (minimum–maximum) and interquartile range (IQR). Comparisons among groups for normally distributed continuous variables were conducted using one-way analysis of variance (ANOVA), followed by Bonferroni post hoc tests. For non-normally distributed variables, the Kruskal–Wallis H test was employed, and pairwise group comparisons were performed using Bonferroni-adjusted Mann–Whitney U tests. Because the evaluated biomarkers were selected a priori on the basis of biological relevance and prior literature, the analyses were considered hypothesis-driven rather than purely exploratory. Group differences were first assessed using omnibus tests, and post hoc pairwise comparisons were adjusted using Bonferroni correction where appropriate. Therefore, raw *p* values from the primary analyses are reported. Moreover, because the subgroup sample sizes were modest, post hoc power analyses were conducted for the GA and treatment-response comparisons using two-sided two-sample tests at α = 0.05. Effect sizes were expressed as Cohen’s d (with Hedges’ correction for small-sample bias).

To further assess whether the inflammatory indices were independently associated with wet AMD, binary logistic regression analyses were performed using AMD status as the dependent variable, adjusting for available confounders, namely age and sex. The most discriminative parameters were then evaluated using ROC curve analysis in both the dry AMD vs. control and wet AMD vs. control groups.

A *p*-value of <0.05 was considered indicative of statistical significance.

## 3. Results

A total of 54 patients with dry AMD, 53 with wet AMD, and 55 healthy controls were included in the study. There were no statistically significant differences among the three groups in terms of age (*p* = 0.088) or sex distribution (*p* = 0.994).

Neutrophil count was significantly higher in the wet AMD group compared to controls (*p* = 0.013), and RDW values were elevated in both dry (*p* = 0.005) and wet AMD groups (*p* = 0.033) compared to controls. Monocyte counts were higher in the wet AMD group compared to both dry AMD (*p* = 0.013) and controls (*p* = 0.046), and platelet count was significantly increased in the wet AMD group compared to the control group (*p* = 0.028). The comparative analysis of hematological parameters among the three groups is summarized in Table 1.

**Figure 1:**
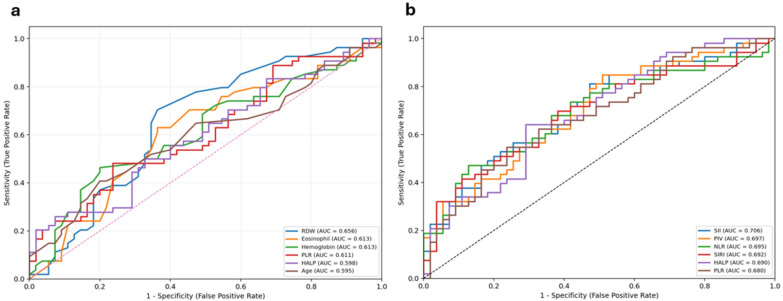
Receiver operating characteristic (ROC) curves of composite inflammatory indices for discriminating dry (**a**) and wet (**b**) AMD from control subjects.

Compared to controls, the wet AMD group had significantly higher values of NLR (*p* < 0.001), PLR (*p* = 0.001), SII (*p* < 0.001), SIRI (*p* < 0.001), and PIV (*p* < 0.001), while the HALP index was significantly lower (*p* < 0.001). Post hoc analysis revealed that NLR, SII, SIRI, and PIV were significantly higher in the wet AMD group compared to both the dry AMD and control groups. HALP was significantly lower in the wet AMD group compared to both groups, while PLR was significantly elevated in both AMD subtypes compared to controls. Additionally, SIRI (*p* = 0.041) and PIV (*p* = 0.029) values were significantly higher in wet AMD compared to dry AMD. The distribution of systemic inflammatory indices across the study groups is presented in Table 2. Moreover, ROC curve analysis, given in Figure 1, was performed to assess the discriminatory performance of the top six candidate variables for dry AMD (1a) and wet AMD (1b) compared to the healthy controls. For dry AMD, among the evaluated markers, RDW showed the highest area under the curve (AUC = 0.656), followed by eosinophil count (AUC = 0.613), hemoglobin (AUC = 0.613), PLR (AUC = 0.611), HALP (AUC = 0.598), and age (AUC = 0.595). Overall, these AUC values indicate limited discriminatory ability. For wet AMD, among the evaluated markers, SII showed the highest area under the curve (AUC = 0.706), followed by PIV (AUC = 0.697), NLR (AUC = 0.695), SIRI (AUC = 0.692), HALP (AUC = 0.690), and PLR (AUC = 0.680). Overall, these AUC values indicate modest discriminatory ability, suggesting that these indices may have adjunctive rather than standalone clinical utility.

To account for potential confounding, multivariable logistic regression analyses were performed with wet AMD status as the dependent variable and age and sex as covariates. Because SIRI and PIV were highly correlated, each inflammatory index was entered into a separate model. After adjustment, higher SIRI (OR per 1-SD increase: 2.69, 95% CI 1.54–4.72, *p* = 0.0005) and higher PIV (OR: 3.06, 95% CI 1.54–6.07, *p* = 0.0014) remained independently associated with wet AMD, whereas higher HALP was inversely associated with wet AMD (OR: 0.43, 95% CI 0.26–0.70, *p* = 0.0007).

Because of the modest sample sizes in the subgroup analyses, post hoc power analysis was performed. The GA subgroup included 22 GA-positive and 32 GA-negative patients, while the treatment-response subgroup included 29 responders and 24 non-responders. With these sample sizes, statistical power was found to be adequate mainly for large effect sizes, whereas it remained limited for moderate effects (approximately 43% power). Effect size and post hoc power estimates for subgroup comparisons are given in Supplementary Table S1.

In the subgroup analysis of the dry AMD group, patients with GA exhibited significantly lower hemoglobin (*p* = 0.002) and erythrocyte counts (*p* = 0.039) compared to those without GA. Post hoc power analysis suggested that the hemoglobin comparison was supported by relatively high observed power (86%), whereas the erythrocyte count comparison showed more modest power (53%). Although white blood cell components and composite inflammatory indices did not differ significantly between these subgroups, the statistical power for most of these comparisons was low, and therefore subtle differences cannot be ruled out. Moreover, no statistically significant differences were identified between TR-positive and TR-negative patients in the wet AMD group concerning any hematological or inflammatory marker. Subgroup analyses comparing patients with GA to those without, as well as those TR-positive and TR-negative in the wet AMD group, are outlined in Table 3.

## 4. Discussion

This study evaluated systemic inflammatory and hematologic biomarkers in patients with dry and wet AMD, with additional subgroup analyses based on the presence of GA in dry AMD and treatment response in wet AMD. Our findings demonstrate that systemic inflammation is more pronounced in patients with wet AMD, while specific hematologic alterations, particularly lower hemoglobin and erythrocyte counts, may be associated with the presence of GA in dry AMD.

Patients with wet AMD exhibited significantly higher neutrophil, monocyte, and platelet counts compared to controls. These elevations were reflected in increased values of composite inflammatory indices such as NLR, PLR, SII, SIRI, and PIV, all of which involve neutrophil-, monocyte-, and platelet-based inflammatory activity. These results are consistent with previous studies suggesting that myeloid cell activation and systemic inflammation contribute to the pathogenesis of neovascular AMD. For instance, Litwinska et al. [14] demonstrated higher neutrophil levels in wet AMD, and Xue et al. [15] reported elevated monocyte counts across various AMD stages. Monocytes and neutrophils are known to secrete cytokines, proteolytic enzymes, and reactive oxygen species, all of which have been implicated in retinal damage and angiogenesis [16,17].

Platelets are also considered active participants in inflammation, due to their role in cytokine release and the recruitment of immune cells [18]. In our study, platelet counts were significantly higher in wet AMD, consistent with prior findings by Sengul et al. [19]. The elevated PLR observed in both dry and wet AMD groups may reflect subclinical systemic inflammation even in early disease stages. Increased thrombopoietin levels and complement-mediated activation have been proposed as underlying mechanisms linking platelet activity to chronic retinal disorders [20,21].

The HALP index, which incorporates hemoglobin, albumin, lymphocyte, and platelet counts, was significantly lower in wet AMD patients compared to controls. Beyond the overall HALP score, the biological relevance of its individual components should also be considered. Hemoglobin reflects systemic oxygen-carrying capacity and may decrease in chronic inflammatory states due to inflammation-mediated suppression of erythropoiesis and dysregulation of iron metabolism. Albumin is widely recognized as a marker of nutritional status and systemic inflammation, and lower albumin levels may reflect chronic inflammatory burden and oxidative stress. In line with this, although serum albumin levels alone did not reach statistical significance in our study, their downward trend in wet AMD supports previous reports describing albumin as a negative acute-phase reactant during chronic inflammatory states [22,23]. Interestingly, albumin has also been identified in proteomic analyses of drusen and Bruch’s membrane, suggesting a potential local ocular role in AMD pathophysiology [24]. Lymphocyte count reflects immune competence and systemic inflammatory balance, whereas lymphopenia has been associated with chronic inflammatory and degenerative conditions. Platelets also contribute to inflammatory signaling, endothelial interaction, and microvascular regulation. Therefore, the HALP score integrates hematologic, nutritional, and inflammatory components into a composite index reflecting systemic inflammatory and metabolic status, which may interact with biological pathways involved in AMD progression, including oxidative stress, complement activation, and chronic low-grade inflammation [25].

Red cell distribution width was elevated in both AMD subtypes compared to controls. RDW is a well-known marker of erythrocyte volume heterogeneity and has been linked to chronic inflammatory conditions and impaired erythropoiesis [25]. Some authors have shown higher RDW levels with AMD, possibly due to oxidative damage affecting red cell membranes and bone marrow function [23]. Our findings reinforce this hypothesis and suggest that red cell indices may serve as complementary biomarkers of systemic involvement in AMD.

In the present study, subgroup analyses were designed to reflect clinically relevant phenotypes within AMD subtypes. In dry AMD, patients were stratified according to the presence of GA, which represents an advanced stage of the disease. Previous studies have suggested that patients with GA may exhibit higher levels of systemic inflammatory markers such as CRP and circulating cytokines, and some reports indicate associations between systemic inflammation and GA presence or progression [26,27]. However, causality remains uncertain, and genetic studies have suggested that systemic inflammatory markers may act more as indicators of inflammatory burden rather than direct drivers of disease progression [28]. In neovascular AMD, patients were categorized according to their response to anti-VEGF therapy, as variability in treatment response represents a major clinical challenge. Emerging evidence suggests that systemic inflammatory activity—including complement pathway activation, pro-inflammatory cytokines, and hematologic inflammatory indices—may be associated with treatment responsiveness, although results across studies remain heterogeneous [29,30].

In subgroup analyses of patients with dry AMD, those with GA had significantly lower hemoglobin and erythrocyte counts compared to those without GA. Although this finding may be biologically relevant, it should be interpreted with caution. Because of the retrospective cross-sectional design, reverse causation cannot be excluded; lower hemoglobin may reflect poorer systemic health, frailty, or chronic comorbidity burden in patients with more advanced disease rather than a direct causal contributor to GA. In addition, despite our exclusion of patients with overt malignancy, hematologic disease, autoimmune disorders, corticosteroid use, and major leukocyte or platelet abnormalities, residual confounding by unmeasured systemic illness remains possible. This is particularly relevant in older adults, in whom chronic inflammation may contribute to anemia through hepcidin-mediated iron sequestration, impaired erythropoietin response, suppressed erythropoiesis, and shortened erythrocyte survival [31]. RDW may also be informative in this context, as elevated RDW is commonly associated with anisocytosis and anemia of chronic inflammation. In our cohort, RDW was numerically higher in the GA subgroup, although this did not reach statistical significance; therefore, the combination of lower hemoglobin/erythrocyte counts and a trend toward higher RDW may be compatible with a chronic inflammatory anemia phenotype, but this interpretation remains speculative [32]. Although white blood cell components and inflammatory indices did not differ significantly between these subgroups, the observed anemia may have pathophysiological relevance. Hypoxia is considered a key driver of RPE and photoreceptor degeneration, particularly in the context of Bruch’s membrane thickening and choriocapillaris loss [33]. Reduced oxygen-carrying capacity in anemic individuals may exacerbate outer retinal ischemia, thereby promoting the development or progression of GA [34]. Unlike some earlier studies that grouped all dry AMD stages together, our study analyzed GA separately, which may account for this novel association.

In contrast, no significant differences in hematologic or inflammatory markers were identified between treatment-responsive and non-responsive patients with wet AMD. While some authors have suggested that systemic inflammation may influence responsiveness to anti-VEGF therapy, our findings did not demonstrate statistically significant differences in systemic hematologic or inflammatory markers between responders and non-responders [35]. However, these results should be interpreted cautiously, as the treatment-response subgroup analysis had limited statistical power, with observed power generally below 20% and reaching a maximum of 41%. Previous studies, including those by Lechner et al. [36], similarly reported no difference in systemic markers between responders and non-responders.

It is important to emphasize that systemic inflammatory indices evaluated in this study are not intended to serve as diagnostic tools for AMD, as the diagnosis of AMD is primarily established through clinical examination and multimodal retinal imaging, including OCT and fluorescein angiography. Rather, these indices may provide complementary information regarding the systemic inflammatory milieu associated with different AMD phenotypes. As these parameters are derived from routine hematological tests, they may represent accessible biomarkers reflecting the systemic inflammatory burden that accompanies AMD pathophysiology. Nevertheless, their clinical utility should be interpreted cautiously, and further prospective studies are required to determine whether such markers may have prognostic or disease-monitoring value.

This study has several limitations. Its retrospective and cross-sectional design precludes causal inference. Systemic inflammatory markers were assessed without direct comparison to intraocular cytokine profiles or imaging-based inflammatory biomarkers. Additionally, subgroup analyses for GA and treatment response were based on modest sample sizes (GA-positive *n* = 22 vs. GA-negative *n* = 32; responders *n* = 29 vs. non-responders *n* = 24), which limited statistical power. Post hoc power estimates indicated that these subgroup comparisons were generally sufficient to detect only large effects, whereas power for moderate effects remained limited. This issue was particularly relevant for the treatment-response analysis, in which observed power was low across all variables. Given the number of biomarkers examined, the possibility of Type I error should be acknowledged. However, the analyses were hypothesis-driven and supported by effect-size estimates and biologically coherent patterns across related inflammatory indices. Lastly, as the study was conducted at a single tertiary referral center, the findings may not be fully generalizable to broader populations.

Despite these limitations, the study has several notable strengths. It is one of the few studies to evaluate a broad spectrum of novel systemic inflammatory indices, including SII, SIRI, PIV, and HALP, in both dry and wet AMD subtypes, while also incorporating subgroup analyses based on GA and TR. The use of routinely available and inexpensive hematological markers enhances the potential clinical applicability of the findings. Moreover, by analyzing these biomarkers in a well-characterized, age- and sex-matched cohort, the study provides valuable insight into the systemic inflammatory landscape of AMD and its subtypes.

## 5. Conclusions

This study demonstrates that patients with wet AMD have significantly elevated systemic inflammatory indices, supporting the role of systemic immune activation in neovascular disease pathogenesis. Hematologic alterations such as lower hemoglobin and erythrocyte counts may be associated with the presence of GA in dry AMD. However, systemic markers were not significantly associated with treatment response in this cohort of wet AMD patients, although this finding should be interpreted cautiously given the limited power of the subgroup analysis. These findings suggest that systemic inflammation contributes differently across AMD subtypes and may serve as a potential biomarker for disease characterization rather than treatment stratification. Future prospective studies involving larger cohorts and longitudinal follow-up are needed to validate the diagnostic and prognostic utility of these biomarkers in clinical practice.

## Figures and Tables

**Table 1:** Comparison of complete blood count parameters and serum albumin levels among dry AMD, wet AMD, and control groups.

	Dry AMD (*n* = 54)	Wet AMD (*n* = 53)	Control (*n* = 55)	*p*_1_	*p*_2_
Hemoglobin (g/dL)	12.69 ± 1.78	12.89 ± 1.41	13.32 ± 1.54	0.110 *	Dry AMD–Wet AMD (*p* = 1.00) Dry AMD–Control (*p* = 0.120) Wet AMD–Control (*p* = 0.489)
Erythrocyte count (×10^6^/μL)	4.47 ± 0.61	4.56 ± 0.48	4.58 ± 0.57	0.559 †	Dry AMD–Wet AMD (*p* = 0.474) Dry AMD–Control (*p* = 0.322) Wet AMD–Control (*p* = 0.612)
Platelet count (×10^3^/μL)	251.75 ± 70.97	263.41 ± 61.66	236.47 ± 48.29	0.105 †	Dry AMD–Wet AMD (*p* = 0.269) Dry AMD–Control (*p* = 0.389) Wet AMD–Control (*p* = 0.028)
Neutrophil count (×10^3^/μL)	4.43 ± 1.50	4.77 ± 1.46	4.40 ± 1.35	0.016 *	Dry AMD–Wet AMD (*p* = 0.590) Dry AMD–Control (*p* = 0.341) Wet AMD–Control (*p* = 0.013)
Lymphocyte count (×10^3^/μL)	2.11 ± 0.75	2.09 ± 0.62	2.32 ± 0.60	0.137 *	Dry AMD–Wet AMD (*p* = 1.000) Dry AMD–Control (*p* = 0.289) Wet AMD–Control (*p* = 0.226)
Eosinophil count (×10^3^/μL)	0.16 ± 0.13	0.20 ± 0.12	0.20 ± 0.12	0.089 †	Dry AMD–Wet AMD (*p* = 0.080) Dry AMD–Control (*p* = 0.041) Wet AMD–Control (*p* = 0.985)
Monocyte count (×10^3^/μL)	0.59 ± 0.21	0.67 ± 0.18	0.60 ± 0.15	0.027 †	Dry AMD–Wet AMD (*p* = 0.013) Dry AMD–Control (*p* = 0.373) Wet AMD–Control (*p* = 0.046)
Leukocyte count (×10^3^/μL)	7.35 ± 1.63	7.79 ± 1.66	7.21 ± 1.32	0.236 †	Dry AMD–Wet AMD (*p* = 0.217) Dry AMD–Control (*p* = 0.811) Wet AMD–Control (*p* = 0.096)
Red cell distribution width (RDW)	14.32 ± 1.28	14.23 ± 1.45	13.73 ± 1.39	0.014 †	Dry AMD–Wet AMD (*p* = 0.620) Dry AMD–Control (*p* = 0.005) Wet AMD–Control (*p* = 0.033)
Albumin (g/dL)	4.26 ± 0.39	4.14 ± 0.34	4.22 ± 0.22	0.148 *	Dry AMD–Wet AMD (*p* = 0.160) Dry AMD–Control (*p* = 1.000) Wet AMD–Control (*p* = 0.670)

Values are presented as mean ± standard deviation. One-way ANOVA test was applied for normally distributed variables (marked with *), and post hoc analysis was performed using the Bonferroni test. Kruskal–Wallis (*p*_1_) test was applied for non-normally distributed variables (marked with †), and pairwise comparisons were conducted using Bonferroni-adjusted Mann–Whitney U tests (*p*_2_).

**Table 2:** Comparison of inflammatory markers among patients with age-related macular degeneration and healthy controls.

	Dry AMD (*n* = 54)	Wet AMD (*n* = 53)	Control (*n* = 55)	*p*_1_	*p*_2_
NLR	2.62 ± 2.17	2.48 ± 1.06	1.83 ± 0.62	0.003	Dry AMD–Wet AMD (*p* = 0.188) Dry AMD–Control (*p* = 0.088) Wet AMD–Control (*p* < 0.001)
PLR	138.85 ± 81.43	135.42 ± 49.64	108.53 ± 39.46	0.005	Dry AMD–Wet AMD (*p* = 0.321) Dry AMD–Control (*p* = 0.045) Wet AMD–Control (*p* = 0.001)
SII	677.24 ± 668.28	660.95 ± 365.36	439.87 ± 187.87	0.001	Dry AMD–Wet AMD (*p* = 0.083) Dry AMD–Control (*p* = 0.100) Wet AMD–Control (*p* < 0.001)
SIRI	1.51 ± 1.25	1.73 ± 1.01	1.12 ± 0.54	0.003	Dry AMD–Wet AMD (*p* = 0.041) Dry AMD–Control (*p* = 0.288) Wet AMD–Control (*p* < 0.001)
PIV	390.88 ± 363.87	471.29 ± 359.35	271.92 ± 149.85	0.001	Dry AMD–Wet AMD (*p* = 0.029) Dry AMD–Control (*p* = 0.232) Wet AMD–Control (*p* < 0.001)
HALP	0.49 ± 0.23	0.44 ± 0.15	0.58 ± 0.22	0.004	Dry AMD–Wet AMD (*p* = 0.197) Dry AMD–Control (*p* = 0.077) Wet AMD–Control (*p* < 0.001)

AMD, age-related macular degeneration; HALP, hemoglobin, albumin, lymphocyte, and platelet index; NLR, neutrophil-to-lymphocyte ratio; PIV, pan-immune–inflammation value; PLR, platelet-to-lymphocyte ratio; SII, systemic immune–inflammation index; SIRI, systemic inflammation response index. *p*_1_ values were obtained using the Kruskal–Wallis test. Pairwise comparisons (*p*_2_) were conducted using Bonferroni-adjusted Mann–Whitney U tests.

**Table 3:** Comparison of hematological and inflammatory parameters according to the presence of geographic atrophy in dry AMD and treatment response in wet AMD.

	Dry AMD			Wet AMD		
	GA (+) (*n* = 22)	GA (−) (*n* = 32)	*p*	TR (+) (*n* = 29)	TR (−) (*n* = 24)	*p*
Hemoglobin (g/dL)	11.8 ± 1.46	13.28 ± 1.76	0.002 *	12.74 ± 1.48	13.07 ± 1.34	0.409 *
Erythrocyte count (×10^6^/μL)	4.27 ± 0.67	4.62 ± 0.53	0.039 *	4.58 ± 0.54	4.55 ± 0.41	0.801 *
Platelet count (×10^3^/μL)	250.31 ± 66.83	252.75 ± 74.73	0.902 *	265.06 ± 68.56	261.41 ± 53.53	0.832 *
Neutrophil count (×10^3^/μL)	4.34 ± 1.64	4.50 ± 1.41	0.712 *	4.85 ± 1.51	4.66 ± 1.42	0.633 *
Lymphocyte count (×10^3^/μL)	1.93 ± 0.73	2.23 ± 0.75	0.150 *	2.16 ± 0.65	2.01 ± 0.59	0.411 †
Eosinophil count (×10^3^/μL)	0.15 ± 0.15	0.17 ± 0.12	0.368 †	0.19 ± 0.11	0.21 ± 0.13	0.518 *
Monocyte count (×10^3^/μL)	0.57 ± 0.19	0.60 ± 0.22	0.609 *	0.71 ± 0.21	0.62 ± 0.14	0.111 †
Leukocyte count (×10^3^/μL)	7.05 ± 1.68	7.55 ± 1.59	0.271 *	7.98 ± 1.79	7.56 ± 1.48	0.364 *
Red cell distribution width (RDW)	14.66 ± 1.28	14.08 ± 1.25	0.099 *	14.32 ± 1.52	14.12 ± 1.39	0.537 †
Albumin (g/dL)	4.33 ± 0.40	4.22 ± 0.37	0.319 *	4.14 ± 0.36	4.14 ± 0.32	0.928 *
NLR	2.72 ± 2.05	2.54 ± 2.27	0.635 †	2.43 ± 1.03	2.54 ± 1.11	0.655 †
PLR	148.58 ± 84.43	132.17 ± 79.97	0.252 †	133.30 ± 55.22	137.98 ± 42.97	0.308 †
SII	731.97 ± 775.69	639.61 ± 593.60	0.686 †	669.24 ± 423.48	650.95 ± 288.59	0.591 †
SIRI	1.55 ± 1.36	1.48 ± 1.20	0.549 †	1.84 ± 1.19	1.59 ± 0.75	0.642 †
PIV	399.95 ± 392.21	384.65 ± 349.36	0.819 †	519.20 ± 437.14	413.40 ± 230.04	0.734 †
HALP	0.42 ± 0.18	0.54 ± 0.24	0.068 *	0.45 ± 0.16	0.42 ± 0.13	0.508 *

AMD, age-related macular degeneration; GA, geographic atrophy; HALP, hemoglobin, albumin, lymphocyte, and platelet index; NLR, neutrophil-to-lymphocyte ratio; PIV, pan-immune–inflammation value; PLR, platelet-to-lymphocyte ratio; SII, systemic immune–inflammation index; SIRI, systemic inflammation response index; TR, treatment response. Values are presented as mean ± standard deviation. Independent samples *t*-test was used for variables with normal distribution (indicated with *), while the Mann–Whitney U test was applied for non-normally distributed variables (indicated with †).

## Data Availability

The datasets used and/or analysed during the current study are available from the corresponding author on reasonable request.

## Supplementary Materials

The following supporting information can be downloaded at: https://www.mdpi.com/article/10.3390/diagnostics16081144/s1, Supplementary Table S1: Effect size and post hoc power estimates for subgroup comparisons.

## Abbreviations

The following abbreviations are used in this manuscript:

AMD	Age-related macular degeneration
GA	Geographic atrophy
cRORA	Complete retinal pigment epithelium and outer retinal atrophy
TR	Treatment response
NLR	Neutrophil-to-lymphocyte ratio
PLR	Platelet-to-lymphocyte ratio
SII	Systemic immune–inflammation index
SIRI	Systemic inflammation response index
PIV	Pan-immune–inflammation value
HALP	Hemoglobin, albumin, lymphocyte, and platelet index
RDW	Red cell distribution width
CBC	Complete blood count
anti-VEGF	Anti-vascular endothelial growth factor
