# Supplementary material for: The Role of Systemic Inflammation in Age-Related Macular Degeneration Subtypes: Exploring Novel Biomarkers
Source: Diagnostics (Basel). 2026 Apr 11;16(8):1144. doi: 10.3390/diagnostics16081144 (PMC13114605; doi:10.3390/diagnostics16081144)
Supplement: Supplementary file 1 [file diagnostics-16-01144-s001.zip › diagnostics-4147160-supplementary.pdf]

Supplementary Table

Table S1. Effect size and post-hoc power estimates for subgroup comparisons in dry AMD and wet AMD patients [geographic atrophy: GA-positive (n=22) vs GA-negative (n=32); treatment response: responders (n=29) vs non-responders (n=24)].

	**Geographic Atrophy**		**Treatment Response**	
**Variable**	**Hedges' g**	**Observed Power (%)**	**Hedges' g**	**Observed Power (%)**
**Age**	0.71	71	-0.01	5
**Erythrocyte count**	-0.58	53	0.07	6
**Leukocyte count**	-0.30	19	0.25	14
**Hemoglobin**	-0.86	86	-0.23	13
**Neutrophil**	-0.10	6	0.13	8
**Lymphocyte**	-0.40	29	-0.24	14
**Monocyte**	-0.14	8	0.49	41
**Eosinophil**	-0.10	6	-0.13	8
**Platelet**	-0.03	5	0.10	7
**RDW**	0.46	37	0.17	10
**Albumin**	0.27	16	-0.08	6
**NLR**	0.08	6	0.15	9
**PLR**	0.20	11	0.12	8
**SII**	0.14	8	0.08	6
**SIRI**	0.06	5	0.25	14
**PIV**	0.04	5	0.30	18
**HALP**	-0.51	44	-0.16	9

Data are presented as Hedges' g effect sizes and observed post-hoc power for the subgroup comparisons. Power was calculated using two-sided independent-samples testing at $\alpha = 0.05$. Negative values of Hedges' g indicate lower values in GA-positive patients or responders compared with their respective reference groups.
